# Transcriptomic changes induced by acute ozone in resistant and sensitive *Medicago truncatula *accessions

**DOI:** 10.1186/1471-2229-8-46

**Published:** 2008-04-23

**Authors:** Michael C Puckette, Yuhong Tang, Ramamurthy Mahalingam

**Affiliations:** 1 246 Noble Research Center, Department of Biochemistry and Molecular Biology, Oklahoma State University, Stillwater, OK, USA; 2 The Samuel Roberts Noble Foundation Inc., Plant Biology Division, Ardmore, OK, USA

## Abstract

**Background:**

Tropospheric ozone, the most abundant air pollutant is detrimental to plant and animal health including humans. In sensitive plant species even a few hours of exposure to this potent oxidant (200–300 nL. L^-1^) leads to severe oxidative stress that manifests as visible cell death. In resistant plants usually no visible symptoms are observed on exposure to similar ozone concentrations. Naturally occurring variability to acute ozone in plants provides a valuable resource for examining molecular basis of the differences in responses to ozone. From our earlier study in *Medicago truncatula*, we have identified cultivar Jemalong is ozone sensitive and PI 464815 (JE154) is an ozone-resistant accession. Analyses of transcriptome changes in ozone-sensitive and resistant accession will provide important clues for understanding the molecular changes governing the plant responses to ozone.

**Results:**

Acute ozone treatment (300 nL L^-1 ^for six hours) led to a reactive oxygen species (ROS) burst in sensitive Jemalong six hours post-fumigation. In resistant JE154 increase in ROS levels was much reduced compared to Jemalong. Based on the results of ROS profiling, time points for microarray analysis were one hour into the ozone treatment, end of treatment and onset of an ozone-induced ROS burst at 12 hours. Replicated temporal transcriptome analysis in these two accessions using 17 K oligonucleotide arrays revealed more than 2000 genes were differentially expressed. Significantly enriched gene ontologies (GOs) were identified using the Cluster Enrichment analysis program. A striking finding was the alacrity of JE154 in altering its gene expression patterns in response to ozone, in stark contrast to delayed transcriptional response of Jemalong. GOs involved in signaling, hormonal pathways, antioxidants and secondary metabolism were altered in both accessions. However, the repertoire of genes responding in each of these categories was different between the two accessions. Real-time PCR analysis confirmed the differential expression patterns of a subset of these genes.

**Conclusion:**

This study provided a cogent view of the unique and shared transcriptional responses in an ozone-resistant and sensitive accession that exemplifies the complexity of oxidative signaling in plants. Based on this study, and supporting literature in Arabidopsis we speculate that plants sensitive to acute ozone are impaired in perception of the initial signals generated by the action of this oxidant. This in turn leads to a delayed transcriptional response in the ozone sensitive plants. In resistant plants rapid and sustained activation of several signaling pathways enables the deployment of multiple mechanisms for minimizing the toxicity effect of this reactive molecule.

## Background

Ozone, a major component of smog in the troposphere, is hazardous to life due to its oxidative nature [1,2]. In ozone-sensitive plants acute exposure to this toxic pollutant leads to necrosis, caused by uncontrolled death of mesophyll cells [3,4]. In resistant plants of the same species there is no visible cell death on exposure to similar ozone concentrations. The physiological parameters especially stomatal conductance is regarded as an important factor determining plant sensitivity to ozone, since it determines how much ozone can enter into the cells [5,6]. However, several studies have indicated a poor correlation between ozone sensitivity and stomatal conductance in various plant species [7-9].

Ozone rapidly degrades into various ROS species at the cell wall interface [10-12]. A biochemical marker for plant sensitivity to ozone is an apoplastic ROS burst after ozone treatment, which is absent or reduced in ozone-resistant plants [12-14]. The role of ROS as a signaling molecule is gaining increasing attention [15-20]. Consequently, plant responses to ozone must be examined in the context that ROS can be cytotoxic at high concentrations and act as key signaling molecules at lower concentrations [21-24].

Alterations in ROS metabolism due to ozone will impact the overall plant redox status [24-26]. Changes in the antioxidants such as ascorbate and glutathione, key components of redox signaling, have been reported in response to ozone [4,8,27-34]. Given that apoplastic ascorbate forms the first line of defense against ozone, differences in ascorbate level have been suggested to be a major factor in determining plant resistance or sensitivity to ozone [35]. This is supported by Arabidopsis *vtc1 *mutant, which has lowered ascorbate levels and is sensitive to ozone [29]. Since ascorbate is an important signal transducing molecule [36] extensive reprogramming of gene expression has been documented in plants with lower levels of this antioxidant [37]. Similarly, interaction of ozone with apoplastic ascorbate could lead to massive transcriptome changes.

The phytohormones salicylic acid (SA) and ethylene (ET) are important second messengers of oxidative stress signaling, involved in lesion initiation and propagation during ozone mediated cell death [5,38-44]. Jasmonic acid (JA) plays an important role in PCD containment by down regulating both SA and ET [40]. Arabidopsis ecotypes and mutants with higher intrinsic levels of ethylene have been reported to be sensitive to ozone [42,43]. Thus naturally occurring variability to ozone in various plant species may be due to intrinsic differences in phytohormone levels.

There are several reports examining changes in plant gene expression in response to ozone using microarrays [4,45-50]. Most of these studies were focused on either an ozone-tolerant or an ozone-sensitive line, or examined changes in gene expression at one time point. Cross-comparisons between these studies are hampered by the differences in ozone treatment conditions, microarray platforms and plant species. In this study we have performed time course analysis of changes in gene expression in response to acute ozone treatment using the ozone-sensitive Jemalong and ozone-resistant JE154 accession. Time points selected were one, six, and twelve hours after treatment initiation. We present the results of this microarray analysis and discuss important gene ontologies (GOs) that are unique to each *M. truncatula *accession and the possible roles of these genes in ozone/ROS signaling.

## Results

### ROS profiles in ozone treated M truncatula accessions

**Figure 1:**
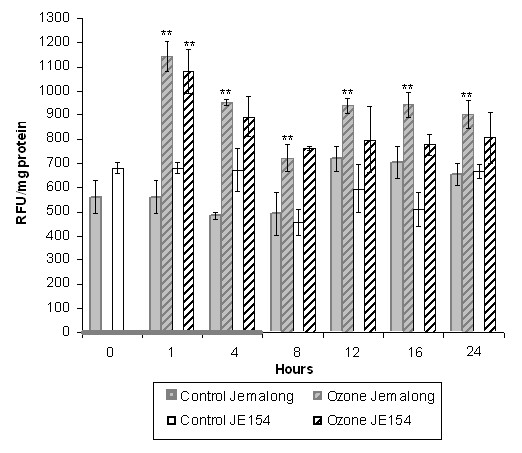
**Total ROS levels in *M. truncatula *accessions**. Leaf samples were taken at 0, 1, 4, 8, 12, 16, and 24 hours. Gray bar on x-axis represents ozone treatment for six hours. ROS levels are reported in Relative Fluorescence Units per mg protein. ** P < 0.01 for significance of total ROS change in ozone treated plants as determined by Student t-test.

ROS analysis of both accessions showed "ozone-derived" ROS peaks at one hour during fumigation (Figure 1). Despite lower stomatal conductivity in JE154 compared with Jemalong [8] this data demonstrated that ozone entry and initial reactions were similar between the two accessions. A distinct "plant-derived" ROS burst was observed in Jemalong 12 hours after initiation of treatment (Figure 1). High levels of ROS were maintained in Jemalong up to 24 hours after treatment initiation. ROS levels in JE154 post-ozone fumigation were slightly higher than the corresponding controls but this was not statistically significant. An interesting observation was the innately higher levels of ROS in JE154 control plants compared to Jemalong. ROS profiling provided a basis for choosing time-points to conduct expression profiling. The one-hour time point was selected since ozone-generated ROS levels were significantly up in both accessions. The six-hour time point represented the end of ozone fumigation. The 12-hour time point marked the start of a sustained plant-derived ROS burst in Jemalong.

### Ozone induced changes in gene expression patterns

**Figure 2:**
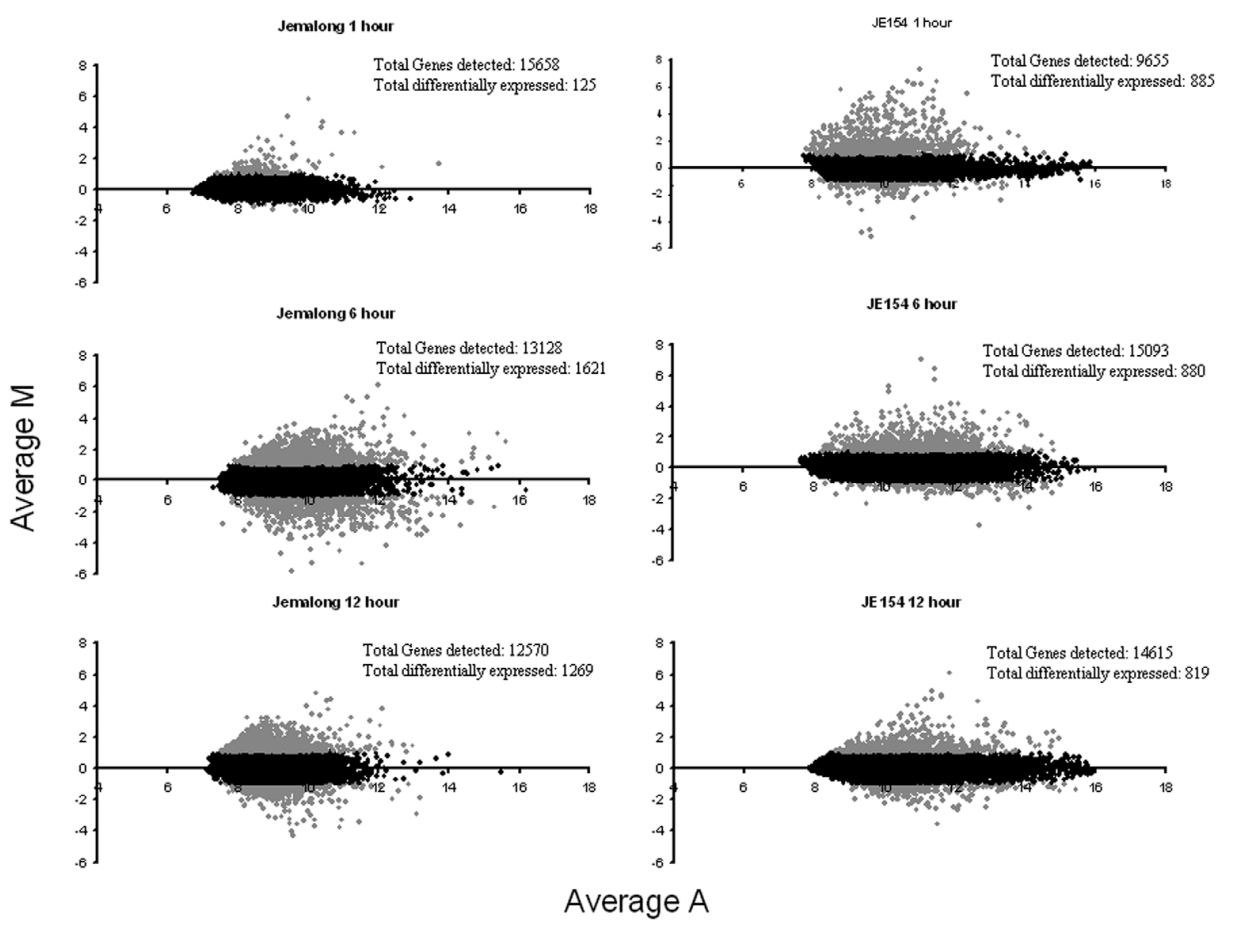
**Ratio-Intensity plots of microarray data in *M. truncatula *accessions**. The M (magnitude) value represents the log2 ratio of change in gene expression of ozone: control. The A (abundance) value represents the log10 value of the product of ozone and control background-corrected hybridization intensity values.

**Figure 3:**
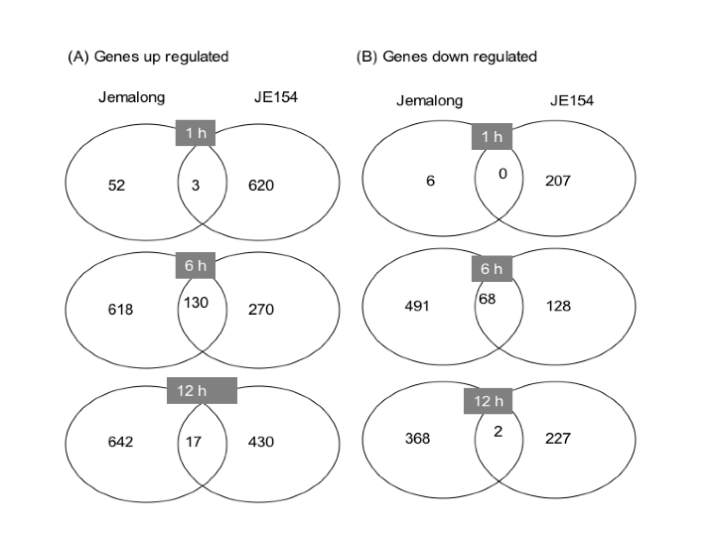
Venn diagram representation of differentially expressed acute ozone responsive genes at different time points in *M truncatula *accessions.

Overall correlations of microarray data from the biological replicates ranged between 0.7–0.87. In JE154, 2102 genes were differentially expressed across all three-time points in response to ozone, while in Jemalong 2397 genes were classified as ozone-responsive [see Additional files 1, 2, 3, 4, 5, 6]. Though the total numbers of genes that were differentially expressed were similar between the two accessions, remarkable differences were observed at the three tested time points. The ratio-intensity plots showed the marked contrast between the two accessions in their temporal patterns of gene expression both in magnitude and direction (Figure 2). In Jemalong, the patterns of changes for both the up and down regulated genes were similar. Early during the ozone treatment there were few changes, while maximal changes in transcriptome were observed at the end of treatment. Number of genes differentially expressed post-treatment was slightly lower compared to the six-hour time point (Figure 3). In JE154, the changes in number of induced genes was opposite to that in Jemalong, with maximal induced genes observed early in treatment and fewer changes at the end of ozone treatment. Like in Jemalong, the number of induced genes increased post-ozone treatment. These contrasting patterns for induced genes may be an important factor contributing to the differences in ozone related phenotypes between these accessions. In JE154 the number of down regulated genes was similar across the three time points (Figure 3).

Commonly differentially expressed genes between accessions were maximal at the end of treatment. A similar pattern was reported in a study of ozone responsive genes in sensitive and resistant pepper cultivars [46]. Despite larger numbers of induced and repressed genes at the 12 hour time point (in comparison with 6 h time point) in JE154, the overlap with the differentially expressed genes of Jemalong at this time point was very low (Figure 3). This suggested that post-ozone treatment different signaling pathways were altered in the two accessions and may be crucial for manifesting the ozone phenotype.

### Gene ontologies of ozone responsive genes

In order to gain insight into the biological significance of genes differentially expressed between accessions we examined GOs using Cluster Enrichment Analysis (CLENCH) [51]. Non-overlapping GOs containing three or more genes are shown in Table 1. All the three levels of GO classification (Process, Function and Compartment) were important in identifying ozone responsive genes. In JE154, 17 GOs were identified as significant one hour into treatment (Table 1). At six hour there was 35 GOs that were significantly overrepresented in Jemalong. This was consistent with the fact that the largest number of differentially expressed genes was identified in this time point. At 12 hours in JE154, 14 unique GO's were upregulated and four were down regulated. In Jemalong the opposite pattern was observed at 12 hours, wherein 11 GOs were repressed and five were up regulated. Despite 33% more genes induced at 12 h in Jemalong compared with JE154, the number of identified GOs was very low, indicating an uncoordinated response in the sensitive plant. Four GOs in the set of induced genes (Response to wounding, response to SA, response to UV, multidrug transport) were common to all time-points in JE154, while none met this criterion in Jemalong. Five GOs in the set of induced genes (Defense response, Response to JA, Response to oxidative stress, Cellulose and pectin containing cell wall, ER) were common to two time points in JE154. Among genes repressed in Jemalong, the GO categories, response to JA and nutrient reservoir activity were enriched at six and 12 hours (Table 1).

**Table 1:** Significant gene ontology categories of ozone-responsive genes in *M truncatula *determined by Cluster Enrichment analysis

Gene Ontology		JE154			Jemalong		
		
	Cat.	1	6	12	1	6	12
Multidrug transport	P	+	+	+		+	
Response to SA	P	+	+	+			-
Response to UV	P	+	+	+			
Response to wounding	P	+	+	+		+/-	
Response to JA	P	+	-	+		-	-
Defense Response	P	+		+			
ATPase activity	F	+					
Calcium ion binding	F	+					
Calmodulin binding	F	+					
Hydrolase activity	F	+					
Kinase activity	F	+					
Sugar porter activity	F	+					
Transcriptional repressor	F	+					
Vacoular membrane	C	+					
Carboydrate metabolic process	P	-					
Transferase activity	F	-					
Translation initiation	P	-					
ER	C		+	+		+	
Response to oxidative stress	P		+	+		+	-
Cellulose and pectin containing cell wall	C		+	+			
Ubiquitination	P		+				
Chloroplast	C		-			-	
Peptidyl-pro-isomerase activity	F		-			-	
Photosynthesis	P		-			-	+
Response to light stimulus	P		-				
Cytosol	C		+			+	-
Phenylpropanoid biosynthesis	P		+			+	
Response to heat	P		+			+	
Senescence	P		+			+	
Copper ion binding	F		-			+	
Toxin catabolism	P		-			+	
AMP binding	F					+	
Cell wall constituent	F					+	
ESCRT III complex	C					+	
Lignin biosynthesis	P					+	
Peroxidase activity	F					+	-
Response to cold	P					+	
Response to fungus	P					+	
Response to salt stress	P					+	
Response to virus	P					+	
Peptidase activity	F			+		-	
Response to light	P			+		-	
Chlorophyll biosynthesis	P					-	
Embryonic development	P					-	
FK506 binding	F					-	
GA mediated signaling	P					-	
Glucan metabolic process	P					-	
Lipoxygenase activity	F					-	
Metal ion binding	F					-	+
Nutrient reservoir activity	F					-	-
Photorespiration	P					-	
Starch biosynthesis	P					-	
Flavonoid biosynthesis	P			+			
Isoprenoid biosynthesis	P			+			
JA biosynthesis	P			+			
Oligopeptide transport	P			+			
Protein folding	P			+			
Sucrose biosynthesis	P			+			
UDP-glycosyltransferase activity	F			+			
Water deprivation	P			+			
Glutathione transferase activity	F			-			
Response to biotic stress	P			-			
Ca ion binding	F						-
Chromosome organization	P						+
Development	P						-
Light harvesting complex	C						+
Protein myristoylation	P						-
Response to ethylene	P						-
Ubiquitin thiolesterase activity	F						+
Unfolded protein binding	F						-

1,6 and 12 refers to the time points in hours at which the GO's were determined to be significant by CLENCH program. Only those GOs that had 3 or more genes are listed in this table.

Abbreviations: Cat. – GO category; P – process; F – function; C – compartment; + indicates that the genes belonging to that GO category were up regulated and - indicates genes in that GO category were down regulated.

### GOs with contrasting responses in JE154 and Jemalong

#### Response to oxidative stress

Seven of the nine genes belonging to GO – response to oxidative stress, were oppositely regulated between the two accessions at the 12 h time point. This included HSP17.6, peroxidase73, calreticulin, isoflavone reductase, chalcone synthase, 2-alkenal reductase and SAG21, whose expression were up regulated in JE154 but were repressed in Jemalong. Induction of peroxidases and 2-alkenal reductase in JE154 indicates the resistant accession was pro-active in reducing oxidative damage. The down regulation of these genes in Jemalong could lead to the build up of ROS and subsequent oxidative cell death.

#### Response to SA and JA

**Figure 4:**
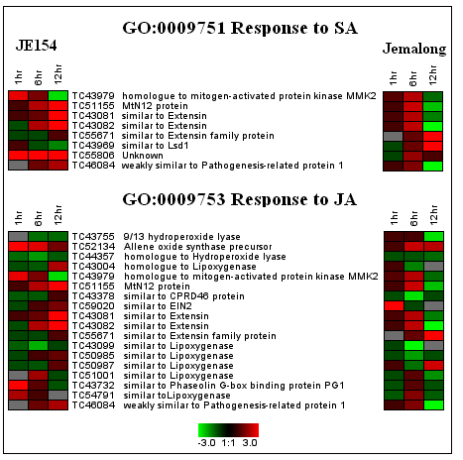
**Heat map view of salicylic acid and jasmonic acid responsive genes differentially expressed in response to acute ozone in *M truncatula *accessions**. Red indicates induction while green color represents repression of gene expression and black indicates no change in expression.

CLENCH analysis identified several GOs associated with phytohormones in both JE154 and Jemalong. Significant up regulation of SA and JA responsive genes were observed at the one-hour time point only in JE154 (Figure 4). We speculate that JE154 may rapidly increase its SA levels due to higher intrinsic ROS compared with Jemalong (Figure 1). This is further supported by the observation that ROS levels in both accessions were similar one hour after ozone treatment, yet this GO did not come up in Jemalong. It is also possible that higher levels of ROS in JE154 could lower the threshold level for SA-induced gene induction in this accession, since this GO was also up at six and 12 hour time points.

Genes associated with JA stimulus showed dramatic differences between accessions. JA responsive genes were up in JE154 at the one and 12-hour time points and were down regulated at the six-hour time point. Enrichment of JA biosynthesis genes at 12 hours in JE154 indicated that this phytohormone could be playing a protective role in containing ROS-induced cell death [52]. JA responsive genes were repressed in Jemalong at the six and 12-hour time points. Genes associated with ethylene signalling were down regulated at the 12 h time point in Jemalong. Thus in Jemalong, SA, JA and ethylene signalling pathways were repressed while in JE154, JA biosynthesis, and SA and JA pathway related genes were induced. The contrasting responses in the hormone signalling genes supports the view that phytohormones play an important role in determining plant responses to ozone [5,40,53].

### Flavonoid Pathway

**Figure 5:**
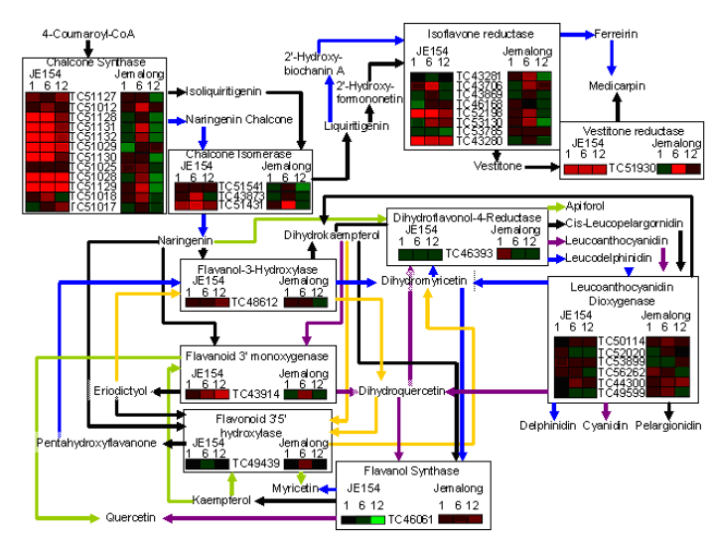
**Representation of flavonoid pathway with cognate heat maps of genes from microarray analysis**. For enzymes with multiple substrates and products, colored arrows connect substrates to their corresponding products.

Increase in transcription of genes involved in flavonoid biosynthesis was observed in JE154 across all time points in response to ozone (Figure 5). Jemalong showed up regulation of flavonoid biosynthesis genes only at six hours. Further, levels of up regulation were less than that observed in JE154. We focused on identifying the subsets of flavonoid pathway that were differentially regulated between the two accessions. At the one-hour time point, JE154 up regulated genes associated with pathways leading to medicarpin biosynthesis, while Jemalong down regulated expression of chalcone synthase homologues. At six hours both accessions up regulated a majority of the genes in flavonoid biosynthesis pathway (Figure 5). The notable difference was that JE154 had a greater degree of up regulation in several genes, most notably chalcone synthase homologues. Given the pivotal role of chalcone synthase to the entire pathway, a significant up regulation could eventually lead to a wider variety of flavonoid compounds [54,55].

At twelve hours, JE154 up regulated genes leading to medicarpin synthesis, as well as, dihydrokaempferol and dihydroquercetin (Figure 5). In addition, JE154 down regulated the enzyme flavonol synthase that uses dihydrokaempferol and dihydroquercetin as a substrate for flavonol synthesis [56,57]. With the up regulation of flavonoid-3-monooxygenase, the enzyme responsible for converting dihydrokaempferol to dihydroquercetin [58], at 12 hours in JE154, the data suggested a buildup of dihydroquercetin. In Jemalong we observed a general down regulation of the pathway particularly leading to medicarpin, and up regulation of leucoanthocyanidin dioxygenase homolog, involved in flavonol biosynthesis.

### GOs unique to JE154

#### Isoprene biosynthesis

Isoprenoids have been shown to be important antioxidants in plants especially in response to ozone [59]. At the 12 hour time point in JE154 key genes in the isoprenoid biosynthesis pathway were strongly up regulated. Both cytosolic and plastidial pathway genes for isoprene biosynthesis are up regulated. The first committed enzyme in plastidial Deoxy-xylulose pathway, Deoxy-xylulose-5-phosphate reductase, and the penultimate enzyme, Isopentenyl-di-phosphate synthase, was up regulated. The first committed enzyme in cytosolic mavelonate pathway, 3-hydroxy-3-Methylglutaryl CoA reductase, was also up regulated. Activation of key genes involved in isoprene biosynthesis in both cytosolic and plastidial compartments suggests an important role for isoprenes in resistant response of JE154 to ozone.

### GOs unique to Jemalong

#### Peroxidase activity

At the six-hour time point Jemalong up regulated six different peroxidases suggesting it was taking active measures to reduce the amount of ROS. Interestingly, this GO (comprising of five of the six genes) was down regulated at the 12-hour time point. Consistent with the down regulation of peroxidases, a concomitant increase in ROS levels were observed in Jemalong that was sustained up to 24 hours (Figure 1).

#### Ascorbic acid biosynthesis

At the six hour time point in Jemalong, two important genes in the ascorbic acid biosynthesis, GDP-mannose-1-phosphate guanyl transferase, and GDP-mannose epimerase, were down regulated. At the same time a copper ion binding protein encoding L-ascorbate oxidase was up regulated. In tobacco plants a homolog of this enzyme is localized to apoplast wherein it oxidizes ascorbate leading to enhanced ozone sensitivity [34,36]. We speculate that oxidation of apoplastic ascorbate due to higher levels of ROS and concomitant reduction in the biosynthesis of ascorbate, may contribute to the increased ozone sensitivity in Jemalong.

### Real-time PCR analysis

We examined the changes in gene expression of 15 genes using real-time PCR. Genes were selected based on the changes in gene expression at the 1, 6 or 12-hour time point. Genes unique to Jemalong or to JE154 were selected for this analysis. Four genes from jemalong and 11 genes from JE154 gave consistent results compared with the microarray data (Table 2).

**Table 2:** Real Time PCR analysis of select ozone responsive genes in *M truncatula*

Acc-time	Gene name	Primer sequences	RT FC/Array FC
Jemalong 1 h	Isoflavone Reductase	F-CTGCTAACCTCTTTGCTGGA	1.7/1.1
	TC52198	R-CCAAAGAGATGAACCTTGTC	
Jemalong 6 h	CA dehydrogenase	F-GGGTGAAAGTAGCAAAGGC	16.2/10.5
	TC51691	R-TGTCAGCTCCAAGATCCTCA	
	Vestitone Reductase	F-GAGCTGATCCAGAACGTAA	4.7/7.9
	TC51930	R-GTTGCTTAGATCGGCGTTG	
Jemalong 12 h	SOD	F-GCTGTGGCAGTTCTTGGTAA	3.1/4.1
	TC51579	R-ACAGTGGTTGGACCATTTCC	
JE154 1 h	Vestitone Reductase	F-GAGCTGATCCAGAACGTAA	7.2/4.0
	TC51930	R-GTTGCTTAGATCGGCGTTG	
	GST 14	F-CATCACTGCATGGATGACCA	10.3/11.6
	TC44882	R-CATGAAATGCCTCTCTGCGA	
	Chitinase	F-TTGTGATCCAGCCTCAAACG	1.9/1.5
	TC45276	R-ACCTCCGAGAGATAGCAAC	
JE154 6 h	Vestitone Reductase	F-GAGCTGATCCAGAACGTAA	9.4/3.6
	TC51930	R-GTTGCTTAGATCGGCGTTG	
	GST 14	F-CATCACTGCATGGATGACCA	3.2/10.1
	TC44882	R-CATGAAATGCCTCTCTGCGA	
	Chitinase	F-TTGTGATCCAGCCTCAAACG	6.8/4.2
	TC45276	R-ACCTCCGAGAGATAGCAAC	
JE154 12 h	Vestitone Reductase	F-GAGCTGATCCAGAACGTAA	6.0/4.0
	TC51930	R-GTTGCTTAGATCGGCGTTG	
	GST 14	F-CATCACTGCATGGATGACCA	7.0/3.2
	TC44882	R-CATGAAATGCCTCTCTGCGA	
	Chitinase	F-TTGTGATCCAGCCTCAAACG	18.8/20.3
	TC45276	R-ACCTCCGAGAGATAGCAAC	
	NAD Dehydrogenase	F-TTCGTTGGGACTGGTTCTTG	-3.6/-2.9
	TC47869	R-GCTCCCACGTAAATAAGTAG	
	Peroxidase 2	F-CTGGTGGTCCATCCTATACA	8.5/8.6
	TC43707	R-GAGGGAGCCTTCCATTTAC	

Abbreviations: Acc-time: accession name – time points. F and R in front of the primer sequences refer to the forward and reverse primers, respectively. CA – cinnamyl alcohol; SOD – superoxide dismutase. RT-FC refers to fold change observed in the real time PCR analysis. Array-FC refers to fold change observed in microarray analysis.

## Discussion

ROS bursts are common in plants in response to environmental perturbations and are generated utilizing multiple cellular processes [60-65]. Based on the similarity of the ROS profiles in response to ozone and avirulent pathogens, ozone has been described as a model abiotic elicitor of ROS in plants [66]. Previous work has shown that in ozone sensitive plants there is a plant produced ROS burst post ozone treatment not present in resistant plants of the same species [12-14,41]. Our studies confirm that there is a sustained ROS in sensitive *M truncatula *accession Jemalong, post-ozone treatment. There was a large variation in ROS levels observed at the 12 h time point in resistant JE154. This may be due to sporadic microscopic cell death previously reported in resistant accessions of other plant species [5,67].

Temporal transcriptome analysis of *M truncatula *accessions provided insights into differences in expression and type of genes that are important for ozone resistance or sensitivity. The massive transcriptome changes, especially the large number of transcription factors [see Additional table 7] and signaling genes differentially regulated early could assist JE154 in adapting to stress rapidly. Down regulation of translation initiation process could potentially allow JE154 to tweak its proteome to limit the extent of damage caused by ozone induced ROS (Table 1). On the contrary, a subdued transcriptional response in Jemalong at the early time point indicates a weakness in the perception and/or signaling of ozone-derived ROS, even though ROS levels were similar to that of JE154 at one hour (Figure 1).

We speculate that the early up regulation of kinase activity especially homologs of OXI1 kinase [68], MAPKKK [69] and several calcium/calmodulin dependent protein kinases facilitated reprogramming JE154 transcriptome towards defensive. The consistent up regulation of defense and hormone responsive genes in JE154 may contribute to the observed resistance to ozone. On the contrary, the down regulation of defense response genes and hormone responsive genes in Jemalong, especially at the 12 hour time point when the ROS levels are significantly high, could lead to its susceptibility to ozone induced oxidative stress.

Increased SA accumulation in Arabidopsis and tobacco plants is associated with ozone sensitivity [41,70]. SA potentiates the generation of ROS in photosynthetic tissues during stresses and ROS in turn triggers the production of SA in a feed-forward self-amplifying loop [41,70-72]. Recent studies have shown that in Arabidopsis plants pathogen-induced NADPH oxidase-derived ROS suppressed cell death and was dependent on SA [73]. It is tempting to speculate that in JE154 the ozone-induced ROS and SA could similarly be suppressing the cell death. Down regulation of peroxidase genes in Jemalong at 12-hour time point may lead to build up of ROS. Coupled with the repression of JA signaling pathway, this can potentially lead to spreading of cell death lesions in Jemalong.

Another aspect of the ozone-induced phenotype in Jemalong was extensive chlorosis. In a comparative study of ozone responses in several bean cultivars, high stomatal conductance accompanied by chlorophyll loss was a common feature of sensitive cultivars [74]. Higher stomatal conductance in Jemalong compared with JE154 [8], and down regulation of genes involved in chlorophyll biosynthesis at six hours in Jemalong supports the observed phenotype and is consistent with report of ozone sensitivity in bean cultivars.

Based on comparisons of microarray data, transcriptional responses to ozone and UV-B have been reported as similar [75]. This shared response may be attributable to the production of antioxidant isoflavonoids [76-78]. Given the importance of these legume natural products [79,80], we examined our microarray data focusing on the flavonoid pathway (Figure 5). Differential expression profiles of the flavonoid pathway at the 12 hour time point is vital in view of the differences in properties of dihydroquercitin and quercitin. Dihydroquercetin has been studied for its antioxidant properties in the animal system where it was able to act as an antioxidant with low toxicity [81-84]. It has also been found to inhibit lipid peroxidation in rats [82]. Since JE154 up regulated flavonoid-3-monoxygenase we speculate it may be able to lower the ROS levels at 12 hours through the antioxidant effects of dihydroquercetin. In addition, down regulation of flavonol synthase may aid in preventing buildup of quercetin. Interestingly, dihydroquercetin and quercetin differ in structure by a double bond, but have very different properties in response to cellular stress [85]. In human endothelial cells exposed to heat stress and chemical treatment, quercetin dramatically increased cell death while dihydroquercetin treatment resulted in cell death rates similar to those of control cells [85]. This suggested that the resistance to ozone in JE154 might be aided by its ability to tweak the flavonoid pathway for maximizing antioxidants promoting cell survival.

In JE154 the up regulation of isoprene biosynthetic pathway could be an important factor for limiting the ROS levels post-ozone fumigation. Isoprenes have strong antioxidant role in plants exposed to ozone and reduce lipid peroxidation of cellular membranes, especially the chloroplast membranes [59,86]. Thus the ozone resistance phenotype of JE154 can be attributed to its swiftness in responding to the stress and the diversity of defense, hormone and antioxidant genes it recruits. Initial tardy response to ozone in Jemalong, exacerbated by down regulation of ascorbate biosynthesis and hormone signaling pathways may be contributing to its sensitivity.

## Conclusion

Naturally occurring genetic variation in M truncatula to acute ozone provides a useful resource for molecular genetic analysis of the oxidative signalling pathway. There were significant differences in the ROS levels in response to acute ozone fumigation between the sensitive Jemalong and resistant JE154. The ozone-generated ROS burst during the treatment was similar in both the accessions. However, the ozone–induced ROS burst, six hours after the end of the treatment were different between the two accessions. ROS profiling showed that similar to tobacco, in M truncatula the bi-phasic ROS burst in response to ozone is observed in resistant JE154 but not in the sensitive Jemalong. Secondly, significant differences in the physiologically relevant, in-planta induced ROS in these two accessions provided a compelling rationale for examining the differences in gene expression at this time point.

Using a ozone-resistant accession and a sensitive accession simultaneously for time-course expression profiling was extremely useful for understanding the temporal dynamics of changes in gene expression in response to ozone. The most striking finding was the seemingly naïve response in Jemalong at the early time point compared to massive changes in gene expression in JE154. We have observed a similar response in the ozone-sensitive Ws-0 ecotype of Arabidopsis [4]. This suggests that in the resistant plants, ozone or ozone-induced signalling molecules are perceived rapidly, that in turn leads to activation of the down stream signalling events. A mapping based strategy using these two accessions will aid in the identification of such ozone resistance genes. Further, examination of pairs of ozone-resistant and sensitive lines in other plant species will aid in determining if ozone sensitivity is due to a passive response with reference to changes in gene expression.

In the resistant JE154, transcriptome analysis indicated early and sustained activation of multiple signalling pathways such as phytohormones, antioxidants, defense response and secondary metabolism. Induction of multiple signalling pathways has been reported in the ozone-resistant Columbia ecotype of Arabidopsis [47,49,50]. As suggested above, examination of pairs of resistant and sensitive lines in other plant species will assist in identifying a common set of ozone responsive genes that can be useful for screening germplasms of crop plants for introgressing ozone resistance trait in plant breeding programs.

## Methods

### Plant growth conditions

JE154 and Jemalong were selected for this study based on their divergent responses to ozone stress [8]. Plants were cultivated in Metro Mix 200 (Scotts-Sierra Horticultural Products Company, Marysville, OH) in a growth room under a light source of 100 μmols s^-1 ^m^-2^, with 10 hours of light and 14 hours of darkness and ambient O_3 _levels (25–30 nmol mol^-1^) for fifty days. Plants were maintained in 2-inch pots and each pot had 1–2 plants. Pots were placed on a plastic tray and were watered at 3–5 day intervals with Peters Excel 15-5-15 fertilizer (Scotts-Sierra Horticultural Products Company, Marysville, OH).

### Ozone treatment

Fifty-day-old plants of both JE154 and Jemalong were exposed to 300 nL L^-1 ^of ozone for six hours, between 10:00 AM and 4:00 PM. Control plants were maintained in the growth room under ambient ozone conditions and identical lighting and temperature settings. Plants were selected randomly from three different pots at each time point. At least 3–4 trifoliates were sampled from each plant. Both young and old trifoliates were harvested at 0, 1, 4, 6, 8, 12, 16, and 24 hours after the start of treatment, frozen in liquid nitrogen, and stored at -80°C. Two biological replications were conducted and the leaf samples from corresponding time points were pooled for ROS analysis and microarray experiments.

### ROS quantification

ROS were measured in control and ozone treated leaf samples using a Versa-Fluor Fluorometer (Bio-Rad) as previously described [8]. The experiment was repeated four times and the results averaged.

### Microarrays and hybridizations

Microarray slides containing 70-mer oligonucleotide were obtained from the Samuel Roberts Noble Foundation and are described previously [87].

Trizol reagent (Invitrogen, Carlbad, CA) was used for total RNA isolation from leaf samples at one, six, and 12 hours after the start of ozone treatment. RNA quality was assessed by northern blot analysis using a GAPDH gene specific probe.

Samples were labeled with Cy-3 and Cy-5 dyes by amino-allyl labeling method [49]. In most of the hybridizations the control cDNA was labeled with Cy3 and the ozone-treated sample was labeled with Cy-5. For one technical replicate for each time point in both accessions, control cDNA was labeled with Cy5 and ozone treated sample was labeled with Cy-3 dye. Hybridizations were carried out overnight at 42°C. Slides were washed with 2 × SSC, 0.2% SDS for five minutes, followed by sequential washes in 1 × SSC for three minutes, 0.5 × SSC for two minutes, 1 × SSC for one minute and 0.05 × SSC for 15 seconds. The slides were dried by spinning in a centrifuge at 500 rpm for five minutes and were scanned in an array scanner (ScanArray Express, PerkinElmer Life Sciences, Boston, MA).

### Microarray data analysis

A total of 19 microarray slides were used in this study. For Jemalong four biological replicates were conducted for the one and six-hour time points and three replicates for the 12-hour time point. There were three biological replicates of JE154 hybridizations for the one and six hour time points, and two replications for the 12-hour time points. Scanned images were analyzed using GenePix software (Molecular Devices, Toronto). Gridding was performed utilizing map files supplied by Noble Foundation (Ardmore, OK). Spots showing signal saturation on both dye channels were flagged and removed from analysis. Mean values of signal intensities were used. A background value of 300 was chosen based on the average fluorescence intensities in non-spotted areas of the slides considered for this analysis.

Pre-processed results from GenePix analysis were fed into GenePix auto processor (GPAP) [88], a web-based application utilizing R (language for statistical computing and graphics) and Bioconductor (freely available software for genomics data sets). Local LOWESS normalization was used to balance the mean fluorescence intensities between the green and red channels. Genes showing a two or more fold change in gene expression (P-value < 0.05) were selected based on this program for further detailed analysis.

### Cluster Enrichment analysis

CLENCH 2.0 [51] was used to analyze the microarray data for enriched gene ontology clusters. Since the *M. truncatula *genome is not as well characterized as *A. thaliana*, genes were examined by BLAST search for *A. thaliana *homologues and used in CLENCH analysis. The total gene list comprised of all the genes that were reliably detected on the microarray for each accession and at each time point. Statistically significant up and down regulated genes were analyzed separately in each accession for each time point. Slim-Terms (provided by TAIR), a coarser level view of the complete GO terms, was used in this analysis.

The template for the flavonoid pathway was retrieved from the KEGG database [89]. The pathway was modified and color-coded using Microsoft Paint program. The heat maps derived from the microarray analysis were generated using the Genesis program [90].

### Real-Time PCR analysis

Total RNA isolated for microarray analysis was diluted to approximately 200 ng/uL. One microgram of this total RNA was used for cDNA synthesis, using Superscript reverse transcriptase (Invitrogen), carried out according to the manufacturer's instructions. A 1:10 and a 1:20 dilution of the cDNA were used for the real-time PCR analysis.

Up and down regulated genes in JE154 and Jemalong at different time points were selected for this analysis. Four genes that were equally expressed in both the lines were used as controls. Primers were designed using Primer express program (Applied Biosystems) to amplify 80–100 base pair fragment and the primers used are listed in the Table 2.

Real-time RT-PCR was performed using the iQ SYBR Green kit (Bio-Rad) as follows: for a 25 μL reaction, 12.5 μL of Master mix, 0.75 μL of 10 μM forward and reverse primers and 1 uL of cDNA template were added. Samples were run and analyzed using GeneAmp 7500 Real Time PCR machine using the cycling protocol 95°C 10 min followed by 95°C 15 sec, 52°C 20 sec, and 72°C 45 sec for 40 cycles.

Normalization was done with the control genes using the delta delta-Ct method. The experiment was repeated twice using different cDNA preparations and the average delta delta Ct values of the two replicates were plotted with standard deviations. In order to determine the delta Ct value, averaged Ct values of ozone treated samples were subtracted from the average Ct value of control samples. Fold change was computed by multiplying the delta Ct value by two. To adjust for differences in cDNA concentrations, real time PCR was performed on genes expressed similarly for each of the three time points. To adjust for differences in cDNA concentrations the fold change value generated from these experiments was then subtracted from the fold change of the samples. For Jemalong 1 hour, Jemalong 12 hour, and JE154 6 hour, gene TC56786 was used to establish differences in cDNA concentrations. Gene TC51792 was used to normalize differences in cDNA concentrations in Jemalong 6 hour. Gene TC46232 was used to normalize differences in cDNA concentrations in JE154, 1 hour. Finally an average of two genes, TC46973 and TC52875, which did not change in expression, was used to establish differences in cDNA concentrations in JE154 12 hour time point.

## Authors' contributions

MCP conducted the ROS analysis, RNA isolations, ozone treatment, microarray hybridizations, data analysis, Real-time PCR and drafted the manuscript. YT provided the microarray slides, protocols for hybridizations and provided comments on the manuscript. RM designed the study, conducted the microarray hybridizations and edited the manuscript. All authors read and approved the final manuscript.

## Supplementary Material

Additional file 1 **Microarray data of JE154-1 h time point**. The M and A-values for each gene on the array for the multiple replicates are given. The average M and A-values from the multiple replicates and the associated p-values are also given. Click here for file

Additional file 2 **Microarray data of JE154-6 h time point**. The M and A-values for each gene on the array for the multiple replicates are given. The average M and A-values from the multiple replicates and the associated p-values are also given. Click here for file

Additional file 3 **Microarray data of JE154-12 h time point**. The M and A-values for each gene on the array for the multiple replicates are given. The average M and A-values from the multiple replicates and the associated p-values are also given. Click here for file

Additional file 4 **Microarray data of Jemalong-1 h time point**. The M and A-values for each gene on the array for the multiple replicates are given. The average M and A-values from the multiple replicates and the associated p-values are also given. Click here for file

Additional file 5 **Microarray data of Jemalong-6 h time point**. The M and A-values for each gene on the array for the multiple replicates are given. The average M and A-values from the multiple replicates and the associated p-values are also given. Click here for file

Additional file 6 **Microarray data of Jemalong-12 h time point**. The M and A-values for each gene on the array for the multiple replicates are given. The average M and A-values from the multiple replicates and the associated p-values are also given. Click here for file

Additional file 7 Ozone responsive transcription factor gene families in M truncatula. Click here for file

## References

[B1] Heagle AS (1989). Ozone and crop yield. Annual Review of Phytopathology.

[B2] Murphy JJ, Delucchi MA, McCubbin DR, Kim HJ (1999). The cost of crop damage caused by ozone air pollution from motor vehicles. Journal of Environmental Management.

[B3] Long SP, Naidu SP, Bell JNB, Treshow M (2002). Effects of oxidants at the biochemical, cell and physiological levels, with particular reference to ozone. Air pollution and Plant life.

[B4] Mahalingam R, Jambunathan N, Gunjan SK, Faustin E, Weng H, Ayoubi P (2006). Analysis of oxidative signalling induced by ozone in Arabidopsis thaliana. Plant Cell and Environment.

[B5] Kangasjarvi J, Jaspers P, Kollist H (2005). Signalling and cell death in ozone–exposed plants. Plant Cell and Environment.

[B6] Saji S, Bathula S, Kubo A, Tamaoki M, Kanna M, Aono M, Nakajima N, Nakaji T, Takeda T, Asayama M, Saji H (2008). Disruption of a gene encoding C-4-dicarboxylate transporter-like protein increases ozone sensitivity through deregulation of the stomatal response in Arabidopsis thaliana. Plant and Cell Physiology.

[B7] Karenlampi L, Metsarinne S, Paakkonen E (1998). Stomatal conductance of birch leaves – Plenty of variation in the variable which determines the ozone dose. Chemosphere.

[B8] Puckette MC, Weng H, Mahalingam R (2007). Physiological and biochemical responses to acute ozone-induced oxidative stress in Medicago truncatula. Plant Physiol Biochem.

[B9] Robinson MF, Heath J, Mansfield TA (1998). Disturbances in stomatal behaviour caused by air pollutants. Journal of Experimental Botany.

[B10] Kanofsky JR, Sima PD (1995). Singlet Oxygen Generation from the Reaction of Ozone with Plant-Leaves. Journal of Biological Chemistry.

[B11] Mehlhorn H, Tabner BJ, Wellburn AR (1990). Electron-Spin-Resonance Evidence for the Formation of Free-Radicals in Plants Exposed to Ozone. Physiologia Plantarum.

[B12] Schraudner M, Moeder W, Wiese C, Van Camp W, Inze D, Langebartels C, Sandermann H (1998). Ozone-induced oxidative burst in the ozone biomonitor plant, tobacco Bel W3. Plant Journal.

[B13] Wohlgemuth H, Mittelstrass K, Kschieschan S, Bender J, Weigel HJ, Overmyer K, Kangasjarvi J, Sandermann H, Langebartels C (2002). Activation of an oxidative burst is a general feature of sensitive plants exposed to the air pollutant ozone. Plant Cell and Environment.

[B14] Pellinen R, Palva T, Kangasjarvi J (1999). Subcellular localization of ozoneinduced hydrogen peroxide production in birch (Betula pendula) leaf cells. Plant Journal.

[B15] Apel K, Hirt H (2004). Reactive oxygen species: Metabolism, oxidative stress, and signal transduction. Annual Review of Plant Biology.

[B16] Gadjev I, Vanderauwera S, Gechev TS, Laloi C, Minkov IN, Shulaev V, Apel K, Inze D, Mittler R, Van Breusegem F (2006). Transcriptomic footprints disclose specificity of reactive oxygen species signaling in Arabidopsis. Plant Physiology.

[B17] Gechev TS, Minkov IN, Hille J (2005). Hydrogen peroxide-induced cell death in Arabidopsis: Transcriptional and mutant analysis reveals a role of an oxoglutarate-dependent dioxygenase gene in the cell death process. Iubmb Life.

[B18] Vranova E, Atichartpongkul S, Villarroel R, Van Montagu M, Inze D, Van Camp W (2002). Comprehensive analysis of gene expression in Nicotiana tabacum leaves acclimated to oxidative stress. Proceedings of the National Academy of Sciences of the United States of America.

[B19] Vranova E, Inze D, Van Breusegem F (2002). Signal transduction during oxidative stress. Journal of Experimental Botany.

[B20] Wagner D, Przybyla D, op den Camp R, Kim C, Landgraf F, Lee K, Wursch M, Laloi C, Nater M, Hideg E, Apel K (2004). The genetic basis of singlet oxygen–induced stress response of Arabidopsis thaliana. Science.

[B21] Breusegem F, Dat J (2006). Reactive Oxygen Species in Plant Cell Death. Plant Physiology.

[B22] Carol RJ, Dolan L (2006). The role of reactive oxygen species in cell growth: lessons from root hairs. Journal of Experimental Botany.

[B23] Gechev TS, Van Breusegem F, Stone JM, Denev I, Laloi C (2006). Reactive oxygen species as signals that modulate plant stress responses and programmed cell death. Bioessays.

[B24] Mahalingam R, Fedoroff N (2003). Stress response, cell death and signalling: the many faces of reactive oxygen species. Physiologia Plantarum.

[B25] Foyer CH, Noctor G (2005). Oxidant and antioxidant signalling in plants: a reevaluation of the concept of oxidative stress in a physiological context. Plant Cell and Environment.

[B26] Foyer CH, Noctor G (2005). Redox homeostasis and antioxidant signaling: A metabolic interface between stress perception and physiological responses. Plant Cell.

[B27] Chameides WL (1989). The Chemistry of Ozone Deposition to Plant-Leaves – Role of Ascorbic-Acid. Environmental Science & Technology.

[B28] Chen Z, Gallie DR (2005). Increasing tolerance to ozone by elevating foliar ascorbic acid confers greater protection against ozone than increasing avoidance. Plant Physiology.

[B29] Conklin PL, Williams EH, Last RL (1996). Environmental stress sensitivity of an ascorbic acid-deficient Arabidopsis mutant. Proceedings of the National Academy of Sciences of the United States of America.

[B30] Gomez LD, Noctor G, Knight MR, Foyer CH (2004). Regulation of calcium signalling and gene expression by glutathione. Journal of Experimental Botany.

[B31] Gupta AS, Alscher RG, Mccune D (1991). Response of Photosynthesis and Cellular Antioxidants to Ozone in Populus Leaves. Plant Physiology.

[B32] Mittova V, Theodoulou FL, Kiddle G, Gomez L, Volokita M, Tal M, Foyer CH, Guy M (2003). Coordinate induction of glutathione biosynthesis and glutathione-metabolizing enzymes is correlated with salt tolerance in tomato. Febs Letters.

[B33] Plochl M, Lyons T, Ollerenshaw J, Barnes J (2000). Simulating ozone detoxification in the leaf apoplast through the direct reaction with ascorbate. Planta.

[B34] Sanmartin M, Drogoudi PD, Lyons T, Pateraki I, Barnes J, Kanellis AK (2003). Overexpression of ascorbate oxidase in the apoplast of transgenic tobacco results in altered ascorbate and glutathione redox states and increased sensitivity to ozone. Planta.

[B35] Conklin PL, Barth C (2004). Ascorbic acid, a familiar small molecule intertwined in the response of plants to ozone, pathogens, and the onset of senescence. Plant Cell and Environment.

[B36] Pignocchi C, Foyer CH (2003). Apoplastic ascorbate metabolism and its role in the regulation of cell signalling. Current Opinion in Plant Biology.

[B37] Pastori GM, Kiddle G, Antoniw J, Bernard S, Veljovic-Jovanovic S, Verrier PJ, Noctor G, Foyer CH (2003). Leaf vitamin C contents modulate plant defense transcripts and regulate genes that control development through hormone signaling. Plant Cell.

[B38] Kwak JM, Nguyen V, Schroeder JI (2006). The role of reactive oxygen species in hormonal responses. Plant Physiology.

[B39] Moeder W, Barry CS, Tauriainen AA, Betz C, Tuomainen J, Utriainen M, Grierson D, Sandermann H, Langebartels C, Kangasjarvi J (2002). Ethylene synthesis regulated by biphasic induction of 1-aminocyclopropane-1-carboxylic acid synthase and 1-aminocyclopropane-1-carboxylic acid oxidase genes is required for hydrogen peroxide accumulation and cell death in ozone-exposed tomato. Plant Physiol.

[B40] Overmyer K, Brosche M, Kangasjarvi J (2003). Reactive oxygen species and hormonal control of cell death. Trends in Plant Science.

[B41] Rao MV, Davis KR (1999). Ozone-induced cell death occurs via two distinct mechanisms in Arabidopsis: the role of salicylic acid. Plant Journal.

[B42] Rao MV, Lee H, Davis KR (2002). Ozone-induced ethylene production is dependent on salicylic acid, and both salicylic acid and ethylene act in concert to regulate ozone-induced cell death. Plant Journal.

[B43] Tamaoki M, Matsuyama T, Kanna M, Nakajima N, Kubo A, Aono M, Saji H (2003). Differential ozone sensitivity among Arabidopsis accessions and its relevance to ethylene synthesis. Planta.

[B44] Vahala J, Keinanen M, Schutzendubel A, Polle A, Kangasjarvi J (2003). Differential effects of elevated ozone on two hybrid aspen genotypes predisposed to chronic ozone fumigation. Role of ethylene and salicylic acid. Plant Physiology.

[B45] D'haese D, Horemans N, De Coen W, Guisez Y (2006). Identification of late O-3-responsive genes in Arabidopsis thaliana by cDNA microarray analysis. Physiologia Plantarum.

[B46] Lee S, Yun SC (2006). The ozone stress transcriptome of pepper (Capsicum annuum L.). Mol Cells.

[B47] Ludwikow A, Gallois P, Sadowski J (2004). Ozone-induced oxidative stress in Arabidopsis: Transcription profiling by microarray approach. Cell Mol Biol Lett.

[B48] Mahalingam R, Gomez-Buitrago A, Eckardt N, Shah N, Guevara-Garcia A, Day P, Raina R, Fedoroff NV (2003). Characterizing the stress/defense transcriptome of Arabidopsis. Genome Biology.

[B49] Mahalingam R, Shah N, Scrymgeour A, Fedoroff N (2005). Temporal evolution of the Arabidopsis oxidative stress response. Plant Molecular Biology.

[B50] Tosti N, Pasqualini S, Borgogni A, Ederli L, Falistocco E, Crispi S, Paolocci F (2006). Gene expression profiles of O-3-treated Arabidopsis plants. Plant Cell and Environment.

[B51] Shah NH, Fedoroff NV (2004). CLENCH: a program for calculating Cluster ENriCHment using the Gene Ontology. Bioinformatics.

[B52] Rao MV, Lee H, Creelman RA, Mullet JE, Davis KR (2000). Jasmonic acid signaling modulates ozone-induced hypersensitive cell death. Plant Cell.

[B53] Rao MV, Davis KR (2001). The physiology of ozone induced cell death. Planta.

[B54] Katsuyama Y, Funa N, Miyahisa I, Horinouchi S (2007). Synthesis of unantural flavonoids and stilbenes by exploiting the plant biosynthetic pathway in Escherichia coli. Chemical Biology.

[B55] Verhoeyen ME, Bovy A, Collins G, Muir S, Robinson S, de Vos CHR, Colliver S (2002). Increasing antioxidant levels in tomatoes through modification of the flavonoid biosynthetic pathway. Journal of Experimental Botany.

[B56] Britsch L, Heller W, Grisebach H (1981). Conversion of Flavanone to Flavone, Dihydroflavonol and Flavonol with an Enzyme-System from Cell-Cultures of Parsley. Zeitschrift Fur Naturforschung C-a Journal of Biosciences.

[B57] Sutter a, Grisebach H (1975). Free Reversibility of Udp-Glucose-Flavonol 3-OGlucosyltransferase Reaction. Archives of Biochemistry and Biophysics.

[B58] Hagmann ML, Heller W, Grisebach H (1983). Induction and Characterization of a Microsomal Flavonoid 3'-Hydroxylase from Parsley Cell-Cultures. European Journal of Biochemistry.

[B59] Loreto F, Velikova V (2001). Isoprene produced by leaves protects the photosynthetic apparatus against ozone damage, quenches ozone products, and reduces lipid peroxidation of cellular membranes. Plant Physiology.

[B60] Allan AC, Fluhr R (1997). Two distinct sources of elicited reactive oxygen species in tobacco epidermal cells. Plant Cell.

[B61] Boveris A, Sanchez R, Beconi M (1978). Antimycin- and cyanide-resistant respiration and superoxide anion production in fresh and aged potato tuber mitochondria. FEBS Letters.

[B62] Krieger-Liszkay A (2005). Singlet oxygen production in photosynthesis. Journal of Experimental Botany.

[B63] Moller IM (2001). Plant mitochondria and oxidative stress: Electron transport, NADPH turnover, and metabolism of reactive oxygen species. Annual Review of Plant Physiology and Plant Molecular Biology.

[B64] Purvis A, Shewfelt R, Gegogeine J (1995). Superoxide production by mitochondria isolated from green bell pepper fruit. Physiologia Plantarum.

[B65] Vianello A, Macri F (1991). Generation of superoxide anion and hydrogen peroxide at the surface of plant cells. Journal of Bioenergetics and Biomembranes.

[B66] Rao MV, Koch JR, Davis KR (2000). Ozone: a tool for probing programmed cell death in plants. Plant Molecular Biology.

[B67] Overmyer K, Tuominen H, Kettunen R, Betz C, Langebartels C, Sandermann H, Kangasjarvi J (2000). Ozone-sensitive *Arabidopsis rcd1 *mutant reveals opposite roles for ethylene and jasmonate signaling pathways in regulating superoxide-dependent cell death. Plant Cell.

[B68] Rentel MC, Lecourieux D, Ouaked F, Usher SL, Petersen L, Okamoto H, Knight H, Peck SC, Grierson CS, Hirt H, Knight MR (2004). OXI1 kinase is necessary for oxidative burst-mediated signalling in Arabidopsis. Nature.

[B69] Kovtun Y, Chiu WL, Tena G, Sheen J (2000). Functional analysis of oxidative stress-activated mitogen-activated protein kinase cascade in plants. Proceedings of the National Academy of Sciences of the United States of America.

[B70] Pasqualini S, Antonielli M, Ederli L, Piccioni C, Loreto F (2002). Ozone uptake and its effect on photosynthetic parameters of two tobacco cultivars with contrasting ozone sensitivity. Plant Physiology and Biochemistry.

[B71] Borsani O, Valpuesta V, Botella MA (2001). Evidence for a role of salicylic acid in the oxidative damage generated by NaCl and osmotic stress in Arabidopsis seedlings. Plant Physiology.

[B72] Shah J (2003). The salicylic acid loop in plant defense. Current Opinion in Plant Biology.

[B73] Torres MA, Jones JDG, Dangl JL (2005). Pathogen-induced, NADPH oxidasederived reactive oxygen intermediates suppress spread of cell death in Arabidopsis thaliana. Nature Genetics.

[B74] Guzy MR, Heath RL (1993). Responses to Ozone of Varieties of Common Bean (Phaseolus-Vulgaris L). New Phytologist.

[B75] Tamaoki M, Nakajima N, Kubo A, Aono M, Matsuyama T, Saji H (2003). Transcriptome analysis of O-3-exposed Arabidopsis reveals that multiple signal pathways act mutually antagonistically to induce gene expression. Plant Molecular Biology.

[B76] Chen S, Hwang J, Deng P (1993). Inhibition of NAD(P)H:Quinone Acceptor Oxidoreductase by Flavones: A Structure-Activity Study. Archives of Biochemistry and Biophysics.

[B77] Lee Y, Westphal A, Haan L, Aarts J, Rietjens I, Berkel W (2005). Human NAD(P)H:Quinone oxidoreductase inhibition by flavonoids in living cells. Free Radical Biology and Medicine.

[B78] Treutter D (2006). Significance of flavonoids in plant resistance: a review. Environmental Chemistry Letters.

[B79] Dixon RA, Achnine L, Kota P, Liu CJ, Reddy MSS, Wang LJ (2002). The phenylpropanoid pathway and plant defence – a genomics perspective. Molecular Plant Pathology.

[B80] Dixon RA, Sumner LW (2003). Legume natural products: Understanding and manipulating complex pathways for human and animal health. Plant Physiology.

[B81] Haraguchi H, Mochida Y, Sakai S, Masuda H, Tamura Y, Mizutani K, Tanaka O, Chou W (1996). Protection against oxidative damage by dihydroflavonols in Engelhardtia chrysolepis. Bioscience, Biotechnology, and Biochemistry.

[B82] Kravchenko L, Morozov S, Tutel'yan V (2003). Effects of Flavonoids on the Resistance of Microsomes to Lipid Peroxidation *In Vitro *and *Ex Vivo*. Bulletin of Experimental Biology and Medicine.

[B83] Teselkin I, Zhambalova B, Babenkova I, Tiukavkina N (1996). Antioxidant properties of dihydroquercetin. Biofizika.

[B84] Teselkin Y, Babenkova I, Kolhir V, Baginskaya A, Tjukavkina N, Kolesnik Y, Selivanova I, Eichholz A (2000). Dihydroquercetin as a Means of Antioxidative Defense in Rats with Tetrachloromethane hepatitis. Phytotherapy Research.

[B85] Budagova K, Zhmaeva S, Grigor'ev A, Goncharova A, Kabakov A (2003). Flavonoid Dihydroquercetin, unlike Quercetin Fails to Inhibit Expression of Heat Shock Proteins under Conditions of Cellular Stress. Biochemistry (Mosc).

[B86] Loreto F, Mannozzi M, Maris C, Nascetti P, Ferranti F, Pasqualini S (2001). Ozone quenching properties of isoprene and its antioxidant role in leaves. Plant Physiology.

[B87] Suzuki H, Achnine L, Xu R, Matsuda SP, Dixon RA (2002). A genomics approach to the early stages of triterpene saponin biosynthesis in Medicago truncatula. Plant Journal.

[B88] http://darwin.biochem.okstate.edu/gpap.

[B89] http://www.genome.jp/kegg.

[B90] Sturn A, Quackenbush J, Trajanoski Z (2002). Genesis: cluster analysis of microarray data. Bioinformatics.

