# Supplementary material for: Transcriptomic changes induced by acute ozone in resistant and sensitive Medicago truncatula accessions
Source: BMC Plant Biol. 2008 Apr 23;8:46. doi: 10.1186/1471-2229-8-46 (PMC2395263; doi:10.1186/1471-2229-8-46)
Supplement: Additional file 7 — Ozone responsive transcription factor gene families in M truncatula. [file 1471-2229-8-46-S7.doc]

Additional Table 7. Ozone responsive transcription factor gene families in *M truncatula*

|  | JE154 | | | Jemalong | | |
| --- | --- | --- | --- | --- | --- | --- |
| TF | 1 h | 6 h | 12 h | 1 h | 6 h | 12 h |
| WRKY | 8 | 4 | 3 | ND | 5 | 3 |
| AP2/EREBP | 4 | ND | 3 | 2 | 6 | 3 |
| NAC | 8 | 2 | 2 | ND | 4 | 1 |
| MYB | 5 | 1 | ND | ND | 1 | 4 |
| HSF | 1 | 2 | 3 | ND | 2 | 2 |
| Homeobox | 3 | ND | 4 | ND | 3 | 2 |

TF – Transcription factor; h-Time point in hours; ND – none detected
